# Nitrogen Isotope Fractionation During Archaeal Ammonia Oxidation: Coupled Estimates From Measurements of Residual Ammonium and Accumulated Nitrite

**DOI:** 10.3389/fmicb.2020.01710

**Published:** 2020-07-28

**Authors:** Maria Mooshammer, Ricardo J. E. Alves, Barbara Bayer, Michael Melcher, Michaela Stieglmeier, Lara Jochum, Simon K.-M. R. Rittmann, Margarete Watzka, Christa Schleper, Gerhard J. Herndl, Wolfgang Wanek

**Affiliations:** ^1^Centre for Microbiology and Environmental Systems Science, University of Vienna, Vienna, Austria; ^2^Department of Functional and Evolutionary Ecology, University of Vienna, Vienna, Austria; ^3^LMU – Max von Pettenkofer Institute for Hygiene and Medical Microbiology, Ludwig Maximilian University of Munich, Munich, Germany; ^4^Department of Marine Microbiology and Biogeochemistry, Royal Netherlands Institute for Sea Research (NIOZ), Utrecht University, Utrecht, Netherlands

**Keywords:** ammonia oxidation, nitrification, nitrous oxide, stable isotope fractionation, Thaumarchaeota

## Abstract

The naturally occurring nitrogen (N) isotopes, ^15^N and ^14^N, exhibit different reaction rates during many microbial N transformation processes, which results in N isotope fractionation. Such isotope effects are critical parameters for interpreting natural stable isotope abundances as proxies for biological process rates in the environment across scales. The kinetic isotope effect of ammonia oxidation (AO) to nitrite (NO_2_^–^), performed by ammonia-oxidizing archaea (AOA) and ammonia-oxidizing bacteria (AOB), is generally ascribed to the enzyme ammonia monooxygenase (AMO), which catalyzes the first step in this process. However, the kinetic isotope effect of AMO, or ε_*AMO*_, has been typically determined based on isotope kinetics during product formation (cumulative product, NO_2_^–^) alone, which may have overestimated ε_*AMO*_ due to possible accumulation of chemical intermediates and alternative sinks of ammonia/ammonium (NH_3_/NH_4_^+^). Here, we analyzed ^15^N isotope fractionation during archaeal ammonia oxidation based on both isotopic changes in residual substrate (RS, NH_4_^+^) and cumulative product (CP, NO_2_^–^) pools in pure cultures of the soil strain *Nitrososphaera viennensis* EN76 and in highly enriched cultures of the marine strain *Nitrosopumilus adriaticus* NF5, under non-limiting substrate conditions. We obtained ε_*AMO*_ values of 31.9–33.1‰ for both strains based on RS (δ^15^NH_4_^+^) and showed that estimates based on CP (δ^15^NO_2_^–^) give larger isotope fractionation factors by 6–8‰. Complementary analyses showed that, at the end of the growth period, microbial biomass was ^15^N-enriched (10.1‰), whereas nitrous oxide (N_2_O) was highly ^15^N depleted (−38.1‰) relative to the initial substrate. Although we did not determine the isotope effect of NH_4_^+^ assimilation (biomass formation) and N_2_O production by AOA, our results nevertheless show that the discrepancy between ε_*AMO*_ estimates based on RS and CP might have derived from the incorporation of ^15^N-enriched residual NH_4_^+^ after AMO reaction into microbial biomass and that N_2_O production did not affect isotope fractionation estimates significantly.

## Introduction

Knowledge of natural ^15^N abundances and of nitrogen (N) isotope fractionation effects associated with key microbial N transformation processes has contributed greatly to our understanding of the marine N cycle (Casciotti and Buchwald, 2012; Buchwald and Casciotti, 2013) and of terrestrial gaseous N emissions (Houlton and Bai, 2009), namely atmospheric N_2_O sources and sinks (Yoshida and Toyoda, 2000), and biological N fixation (Vitousek et al., 2013). The oxidation of NH_4_^+^ to NO_2_^–^—the first and rate-limiting step in nitrification—is a central process in the marine and terrestrial N cycles, as well as the major driver of a large N isotope effect that leads to formation of ^15^N-depleted products such as NO, N_2_O, NO_2_^–^, and NO_3_^–^, while residual NH_4_^+^ becomes ^15^N-enriched during that process (Mariotti et al., 1981; Sigman and Casciotti, 2001). A detailed understanding of N isotope effects of the range of N transformation processes is thus critical for adequate biological interpretation of natural ^15^N isotope patterns in the environment (Casciotti, 2016).

Besides the recently discovered comammox bacteria (Daims et al., 2015; van Kessel et al., 2015), ammonia oxidation is catalyzed by both ammonia-oxidizing archaea (AOA) and ammonia-oxidizing bacteria (AOB), with different relative contributions across ecosystems and environmental conditions (Prosser and Nicol, 2012; Prosser et al., 2019). On a cellular level, ammonia oxidation is a multi-step process that comprises different enzymatic reactions and chemical equilibrium processes, which can all contribute to the N isotope fractionation effects inferred from extracellular N pools (Casciotti et al., 2003; Santoro and Casciotti, 2011). The isotopic fractionation effect (ε) of ammonia oxidizers has been typically inferred based on changes in δ^15^N of the cumulative product (CP) NO_2_^–^ (ε_*CP*_), and attributed to the initial enzymatic step catalyzed by the ammonia monooxygenase (AMO) enzyme, defined as ε_*AMO*_. However, ε_*CP*_ estimates reflect the combined fractionation effects of the isotope equilibrium between NH_4_^+^ and NH_3_ [NH_3_, the proposed substrate for ammonia oxidation, is depleted in ^15^N relative to NH_4_^+^ (Hermes et al., 1985)], the AMO-catalyzed reaction, and accumulation of several intermediates derived from subsequent enzymatic processes (Casciotti et al., 2003). For instance, ε_*CP*_ estimates may be affected by the accumulation of essential intermediates, such as hydroxylamine (NH_2_OH) and by the production of gaseous N by-products (nitric oxide, NO; and nitrous oxide, N_2_O), which may represent further ^15^N fractionation steps. Consequently, this could result in a difference of kinetic isotope effect estimates derived from residual substrate (RS) and CP (Casciotti et al., 2003). Not only could these “leakage” processes alter CP-based estimates of ε_*AMO*_, but their different contributions to ammonia utilization and to ε_*CP*_ may also underlie the large differences observed in ε_*AMO*_between ammonia-oxidizing organisms (Mariotti et al., 1981; Yoshida, 1988; Casciotti et al., 2003; Santoro and Casciotti, 2011).

Estimates of isotope effects based on the change in δ^15^NH_4_^+^ (ε_*RS*_) can circumvent many of the expected biases associated with ε_*CP*_, as they are not affected by the multiple subsequent equilibria, enzymatic transformations, and intermediate N pools, as discussed but not quantified previously (Casciotti et al., 2003; Santoro and Casciotti, 2011). However, to our knowledge, only one study has determined the isotope fractionation factors based on concurrent measurements of changes in isotopic composition of RS and CP of ammonia oxidation, namely in cultures of the AOB *Nitrosomonas europaea* (Mariotti et al., 1981). This study found no difference between ε_*RS*_ and ε_*CP*_, suggesting that ammonia oxidation can be effectively regarded as a “one-step process,” where the AMO-catalyzed reaction constitutes the rate-limiting and sole isotope fractionation step. On the other hand, AOB and AOA seem to harbor fundamentally distinct ammonia oxidation pathways and exhibit different yields of gaseous N compounds per mole of NH_4_^+^ consumed (Walker et al., 2010; Kozlowski et al., 2016). Importantly, the enzyme hydroxylamine dehydrogenase (HAO), which performs the second step in ammonia oxidation of AOB, has not been identified in AOA, and thus it remains unclear how NH_2_OH is converted to NO_2_^–^ in AOA (Walker et al., 2010; Kerou et al., 2016). Moreover, a recent study has provided evidence that the bacterial HAO oxidizes NH_2_OH to NO rather than to NO_2_^–^, as generally assumed, with the latter resulting from non-enzymatic oxidation of NO by oxygen (Caranto and Lancaster, 2017). Previous studies have shown that NO is also an essential intermediate in ammonia oxidation by AOA, as their growth and activity is highly sensitive to exposure to an NO scavenger (Shen et al., 2013; Kozlowski et al., 2016).

Here, we tested whether the kinetic isotope effect of archaeal ammonia oxidation based on CP (δ^15^NO_2_^–^) alone might be biased, by comparing the isotope fractionation factors inferred from both RS and CP pools. For this, we determined the kinetic isotope effects during growth of two phylogenetically and ecologically distinct AOA: the axenic strain *Nitrososphaera viennensis* EN76 (Stieglmeier et al., 2014a), isolated from soil, and the highly enriched marine strain *Nitrosopumilus adriaticus* NF5 (Bayer et al., 2016). This is also the first study of ^15^N isotope fractionation of ammonia oxidation by an AOA strain in pure culture. All previous studies of kinetic isotope effects of AOA have been performed with enrichment cultures with varying degrees of enrichment (Santoro and Casciotti, 2011; Nishizawa et al., 2016) and bacterial contaminants that may have contributed to the variation in isotope effects through consumption of and inputs to the same N pools.

## Materials and Methods

Pure cultures of *N. viennensis* EN76 were cultivated in freshwater medium and incubated at 37°C, as described by Tourna et al. (2011). In a first experiment, quadruplicate cultures were supplemented with 1 mM NH_4_^+^ and 0.1 mM pyruvate; in a second experiment, quadruplicate cultures were supplemented with 2 mM NH_4_^+^ and 0.5 mM pyruvate to generate higher cell biomass and sufficient N_2_O concentrations for isotopic analysis, in order to determine their potential effect on ε_*AMO*_. Quadruplicate enrichment cultures of *N. adriaticus* NF5 were cultivated in Synthetic Crenarchaeota Medium (SCM) at 30°C as described by Bayer et al. (2016). The medium was supplemented with 1 mM NH_4_^+^ and 5% (v/v) autoclaved seawater, which was sterile-filtered (0.22 μm GTTP, Millipore). Kanamycin at a final concentration of 100 μg ml^–1^ was used to inhibit bacterial contaminants. At the time of the experiment (January 2013), the enrichment level of strain NF5 was ∼95%, as it contained a heterotrophic non-nitrifying/non-denitrifying contaminant of the alphaproteobacterial species *Oceanicaulis alexandrii* (Bayer et al., 2019).

Ammonia-oxidizing archaea growth was monitored by measuring nitrite production using the Griess method (Hood-Nowotny et al., 2010), coupled to NH_4_^+^ consumption determined using the Berthelot method for *N. viennensis* cultures (Hood-Nowotny et al., 2010) and the o-Phthalaldehyde (OPA) method for *N. adriaticus* cultures (Goyal et al., 1988). δ^15^NH_4_^+^ was quantified by microdiffusion (Sørensen and Jensen, 1991) with subsequent analysis on a continuous-flow isotope ratio mass spectrometer consisting of an elemental analyzer (EA1110, CE Instruments) coupled via a ConFlo III interface (Finnigan MAT, Thermo Fisher Scientific) to the isotope ratio mass spectrometer (IRMS; DeltaPLUS, Finnigan MAT, Thermo Fisher Scientific). δ^15^NO_2_^–^ was determined based on the reduction of NO_2_^–^ to N_2_O by azide under acidified conditions (Lachouani et al., 2010). Concentrations and isotopic ratios of N_2_O were determined using a purge-and-trap GC/IRMS system (PreCon - GasBench II headspace analyzer, Delta Advantage V IRMS; Thermo Fischer Scientific, Vienna, Austria). For NH_4_^+^ and NO_2_^–^ isotope measurements, we included blanks, concentration standards, and isotope standards varying in natural ^15^N abundance together with the samples through the full microdiffusion and azide procedures to allow corrections for blank contribution, incomplete reaction, and procedural isotope fractionation (Lachouani et al., 2010). Nitrogen content and δ^15^N signature of AOA biomass were determined by EA-IRMS as described above. δ^15^N signatures [‰ vs. AIR] were calculated relative to the ratio R (^15^N:^14^N) of the atmospheric N_2_ standard (AIR), as δ^15^N = (*R*_sample_/*R*_standard_− 1) × 1000.

Isotope fractionation factors (ε) were calculated based on the Rayleigh closed system isotope fractionation, based on changes in the isotopic compositions of RS (i.e., NH_4_^+^) and CP (i.e., NO_2_^–^) (Mariotti et al., 1981):

(1) $$10^{3}\,\text{ln}\,\frac{10^{-3}\,\delta_{R\,S}+1}{10^{-3}\,\delta_{S\,0}+1}=\varepsilon \,l\,n\,\left(f\right)$$

(2) $$\delta_{C\,P}-\delta_{S\,0}=-\varepsilon \,f\,\frac{\text{ln}\,\left(f\right)}{\left(1-f\right)},$$

where δ_*S*0_ is δ^15^N of initial NH_4_^+^, δ_*RS*_ is δ^15^NH_4_^+^, δ_*CP*_ is δ^15^NO_2_^–^ and *f* is the fraction of the initial [NH_4_^+^] remaining in the culture. Plots of $10^{3}\,\frac{10^{-3}\,\delta_{R\,S}+1}{10^{-3}\,\delta_{S\,0}+1}$ versus *ln*⁡(*f*) and of δ_*CP*_−δ_*S*0_ versus$f\,\frac{\text{ln}\,\left(f\right)}{\left(1-f\right)}$ yield linear relations, with the slope representing the kinetic isotope effect based on the isotopic change in substrate (ε_*RS*_) and product (ε_*AP*_), respectively. Uncertainties of ε are expressed as standard error of the slope. Differences in isotope fractionation effects between cultures were assessed by testing significant differences between their regression plots, using R (R Development Core Team, 2012).

## Results and Discussion

**FIGURE 1:**
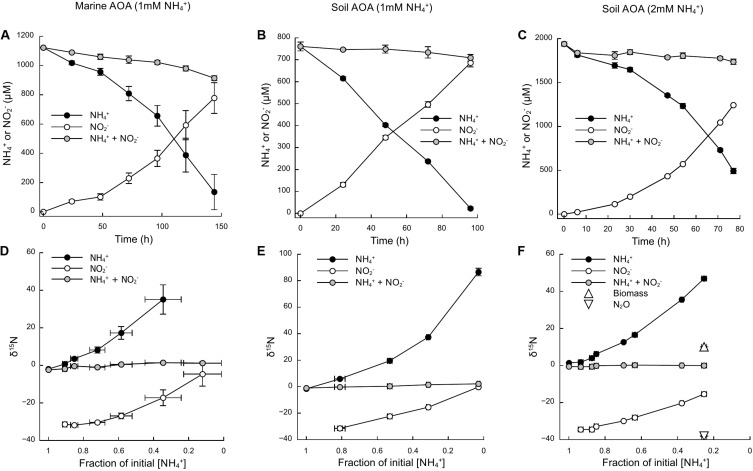
Ammonia oxidation and isotopic signature of NH_4_^+^ and NO_2_^–^ of a marine (*N. adriaticus* NF5 enrichment culture) and a soil (*N. viennensis* EN76) AOA. **(A–C)** Time course of NH_4_^+^ oxidation to NO_2_^–^. **(D–F)** Isotopic composition of NH_4_^+^ and NO_2_^–^ as a function of initial NH_4_^+^ oxidized. Some error bars are smaller than the symbol.

**FIGURE 2:**
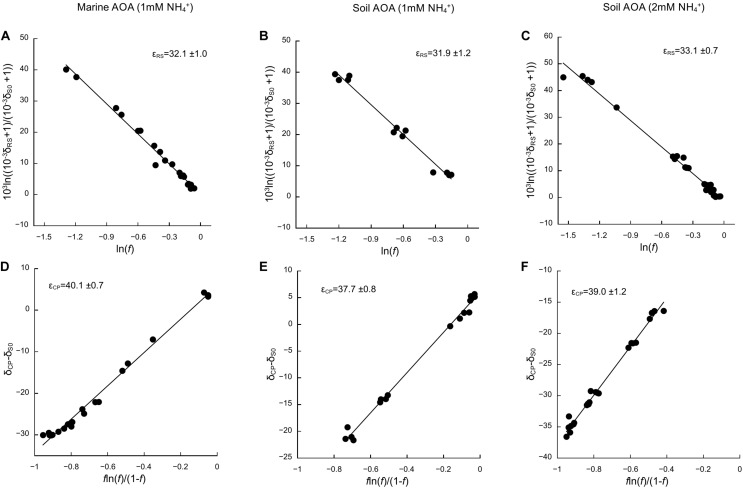
Kinetic isotope effects of the marine AOA *N. adriaticus* and the soil AOA *N. viennensis*. Isotope fractionation factors (ε_*RS*_ and ε_*CP*_) were calculated based on changes in **(A–C)** δ^15^N-NH_4_^+^ (δ_*RS*_) and **(D–F)** δ^15^N-NO_2_^–^ (δ_*CP*_), using linear regressions of $10^{3}\,\frac{10^{-3}\,\delta_{R\,S}+1}{10^{-3}\,\delta_{S\,0}+1}$ versus ln(*f*) and δ_*CP*_−δ_*S*0_ versus $f\,\frac{\text{ln}\,\left(f\right)}{\left(1-f\right)}$, respectively, as described in Mariotti et al. (1981). δ_*S*0_ is δ^15^N of initial NH_4_^+^ and *f* is the fraction of the initial [NH_4_^+^] remaining in the culture. Uncertainties of ε_*RS*_ and ε_*CP*_ are expressed as SE of the slope. We used multiple-point estimates because they have lower uncertainty than single-point analyses.

Based on the oxidation of NH_3_/NH_4_^+^ to NO_2_^–^—a typical proxy for ammonia oxidizer growth, as it strongly correlates with growth rates (Stieglmeier et al., 2014a; Bayer et al., 2016)—all cultures showed growth curves typical for batch cultures of AOA, reaching stationary phase after 7 days for *N. adriaticus*, and after 3–4 days for *N. viennensis* cultures (Figures 1A–C). Nitrogen isotope fractionation was reflected in both the substrate (i.e., NH_4_^+^) and the product (i.e., NO_2_^–^) of ammonia oxidation, and followed typical Rayleigh isotope fractionation kinetics for closed systems (Figures 1D–F): NH_4_^+^ became increasingly ^15^N-enriched with the fraction of NH_4_^+^ oxidized, while NO_2_^–^ was strongly ^15^N-depleted after correction for NO_2_^–^ deriving from the inoculum. With an increasing fraction of NH_4_^+^ oxidized, δ^15^NO_2_^–^ converged toward the isotopic signature of the initial NH_4_^+^. Both *N. adriaticus* and *N. viennensis* (including cultures grown on 1 and 2 mM NH_4_^+^) exhibited ^15^N isotope fractionation factors based on substrate (ε_*RS*_) between 31.9 and 33.1‰, and based on product (ε_*CP*_) between 37.7 and 49.1‰ (Figures 2A–F). We found no significant difference between the isotope fractionation factors of the different AOA cultures studied here based on δ^15^N evolution of the substrate (ε_*RS*_; comparison of slopes, *df* = 2, *F* = 0.519, *p* = 0.598) or the product (ε_*CP*_; comparison of slopes, *df* = 2, *F* = 2.380, *p* = 0.102). The N isotope fractionation factors based on δ^15^NO_2_^–^ (ε_*CP*_) were larger than those based on δ^15^NH_4_^+^ (ε_*RS*_) by 8.0, 5.8, and 5.9‰ for *N. adriaticus*, and for *N. viennensis* grown on 1 mM or 2 mM NH_4_^+^, respectively.

Nitrogen isotope fractionation has been studied in several AOB strains, but only in three marine and one thermophilic AOA enrichment cultures. These AOA enrichment cultures showed average N isotope fractionation factors between 22 and 25‰ at low substrate concentrations, and up to 32.0‰ at higher ammonium concentrations (Santoro and Casciotti, 2011; Nishizawa et al., 2016, measured via the isotopic composition of the product nitrite; see Table 1). These estimates are in the same range as the reported average isotope effects for different AOB strains, i.e., 14–38‰ (Delwiche and Steyn, 1970; Mariotti et al., 1981; Casciotti et al., 2003). ^15^N isotope fractionation factors of *N. viennensis* and *N. adriaticus* are in the upper range, or higher, than those previously reported for AOA, which might be due to the higher ammonia concentrations applied in our study (1–2 mM in our study vs. 200 μM in Nishizawa et al., 2016; 10–75 μM in Santoro and Casciotti, 2011). Previous studies have indicated that higher initial ammonia concentrations lead to more stable and higher ^15^N isotope fractionation (Casciotti et al., 2003; Santoro and Casciotti, 2011).

**TABLE 1:** Compilation of published kinetic isotope effects of AOA and AOB.

**Source**	**AOA/AOB**	**Strain**	**Initial [NH_4_^+^] (mM)**	**Other conditions**	**ε_*RS*_**		**ε_*CP*_**	
					**Mean**	**SD**	**Mean**	**SD**
This study	AOA	*Nitrosopumilus adriaticus* NF5	1		32.1	1.0	40.1	0.7
	AOA	*Nitrososphaera viennensis* EN76	1		31.9	1.2	37.7	0.8
	AOA	*Nitrososphaera viennensis* EN76	2		33.1	0.7	39.0	1.2
Santoro and Casciotti (2011)	AOA	Marine AOA enrichment CN25^†^	0.01–0.075				22	5
		Marine AOA enrichment CN75					21	10
		Marine AOA enrichment CN150					22	5
Nishizawa et al. (2016)	AOA	*Candidatus* Nitrosocaldus sp.	0.2				22.0	5.0
		*Candidatus* Nitrosocaldus sp.	14				24.7	2.1
Mariotti et al. (1981)	AOB	*Nitrosomonas europaea*	4.7–25		34.7	2.5	31.9	6.4
Delwiche and Steyn (1970)	AOB	*Nitrosomonas europaea*					26.0	5.6
Yoshida (1988)	AOB	*Nitrosomonas europaea*	38	pO_2_ low			24.6	
	AOB	*Nitrosomonas europaea*	38	pO_2_ medium			29.0	
	AOB	*Nitrosomonas europaea*	38	pO_2_ high			32.0	
Casciotti et al. (2003)	AOB	*Nitrosomonas marina*	2				14.2	3.6
		*Nitrosomonas sp.* C-113a	2				19.1	1.2
		*Nitrosospira tenuis*	1				24.6	1.4
		*Nitrosomonas eutropha*	1				32.8	1.7
		*Nitrosomonas europaea*	1				38.2	1.6
Casciotti et al. (2010)	AOB	*Nitrosomonas* sp. C-113a	0.005–0.05				30–46	
	AOB	*Nitrosococcus oceani*	0.005–0.05				30–46	
	AOB	*Nitrosospira briensis*	0.005–0.05				30–46	

*^†^Currently designated as *Candidatus* Nitrosopelagicus brevis CN25 (Santoro et al., 2015).*

We also measured ε_*AMO*_ based on changes in δ^15^NH_4_^+^ (i.e., the residual substrate) to both circumvent and assess potential biases associated with estimates based on δ^15^NO_2_^–^ (i.e., the cumulative product). It should be noted, however, that different apparent isotope effects in whole cells may also be observed in the NH_4_^+^ pool, despite constant AMO enzyme-level isotope effects, depending, for example, on the balance between ammonia oxidation rates and ammonia diffusion across the S-layer (i.e., outermost cell envelope component in AOA) (Casciotti et al., 2003; Li et al., 2018). Published models of AOA and AOB metabolism favor the hypothesis of a (pseudo-)periplasmic location of the ammonia oxidation process (Arp and Stein, 2003; Walker et al., 2010; Simon and Klotz, 2013). However, AOA and AOB harbor very distinct NH_3_/NH_4_^+^ transport systems (Offre et al., 2014), whose role in ammonia oxidation and contribution to observed differences in ^15^N isotope fractionation remain unclear (Arp and Stein, 2003). At low ammonia concentrations, ammonia oxidation rates are expected to become limited by NH_4_^+^ transport/NH_3_ diffusion, resulting in the convergence of the isotope effect toward that of NH_4_^+^/NH_3_ equilibrium (if NH_3_ is mainly taken up by the cells) or NH_4_^+^/NH_3_ transport. The NH_4_^+^/NH_3_ equilibrium isotope effect has been estimated to be 19.2‰ in aqueous solution (Hermes et al., 1985), whereas secondary active ammonium (AMT) transporters, which are highly expressed in AOA (Carini et al., 2017), have been shown to exert isotope fractionation of around 13–15‰, due to deprotonation of NH_4_^+^ during transport (Ariz et al., 2018). It is unlikely that ammonia oxidation has been limited by NH_3_ availability in our study, because of the high substrate concentrations used, which are well above the *K*_*m*_ of the AMO of *N. viennensis* (5.4 μM NH_3_ + NH_4_^+^; Kits et al., 2017) and that of the marine strain *Nitrosopumilus maritimus* strain SCM1 (0.13 μM NH_3_ + NH_4_^+^; Martens-Habbena et al., 2009), which is closely related to *N. adriaticus*. Furthermore, Nishizawa et al. (2016) estimated that, when NH_3_ concentrations in the pseudo-periplasm are lower than in the medium under laboratory conditions, cell-specific NH_3_ diffusion rates into the pseudo-periplasm are higher than cell-specific ammonia oxidation rates. It has also been proposed that the charged S-layer proteins of AOA enhance the diffusion of charged solutes, such as NH_4_^+^, which concentrates NH_4_^+^ in the pseudo-periplasmic space near the active site of the AMO (Li et al., 2018), where then the equilibrium reaction between NH_4_^+^ and NH_3_ is relatively fast and considered not to be rate-limiting.

**FIGURE 3:**
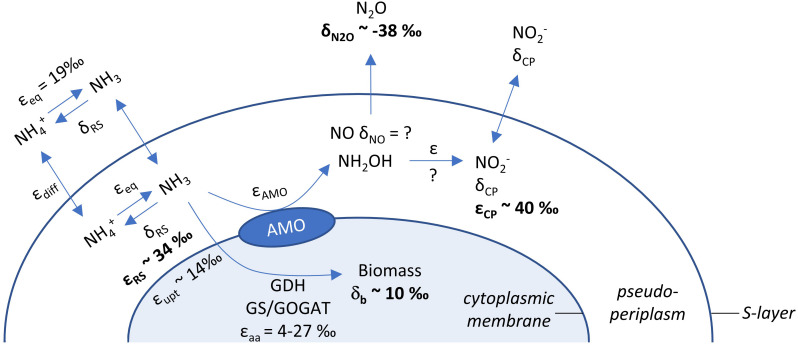
Schematic overview of processes and isotope fractionation effects involved in ammonia oxidation, growth and intermediate formation of the soil AOA *N. viennensis*. δ^15^N values are given for endpoint measurements of N_2_O and biomass, while average kinetic isotope effects of ammonia oxidation are presented for substrate (NH_4_^+^, ε_*RS*_) and product (NO_2_^–^, ε_*CP*_). Literature values for isotope fractionation of NH_3_/NH_4_^+^ equilibration (ε_*eq*_; causing ^15^N depletion of NH_3_), for secondary active NH_4_^+^ uptake (ε_*upt*_) and ammonia assimilation (ε_*aa*_) are presented as well. The identity of the enzyme oxidizing NH_2_OH to NO_2_^–^ and its inherent isotope fractionation are currently unknown for AOA.

Even if ammonia oxidation was not limited by periplasmatic NH_3_ availability, the apparent isotope effect of the AMO can also be underestimated due to concurrent NH_4_^+^ assimilation, which has a smaller isotope effect. This process would alter observed ε_*RS*_ estimates in proportion to the amount of NH_4_^+^ assimilated and the isotope effect for NH_4_^+^ assimilation (4–27‰; Hoch et al., 1992). Therefore, we also measured δ^15^N of the cell biomass at the end of incubation of *N. viennensis* grown on 2 mM NH_4_^+^ (Figures 1F, 3). Although it is impossible to infer directly the contribution of N assimilation to ε_*RS*_ from just one end-point measurement, we propose that N assimilation substantially contributed to the decrease of ε_*RS*_ relative to ε_*CP*_ in our study, as biomass was ^15^N-enriched by ∼10‰ compared to initial NH_4_^+^. Biomass N represented 3.1% (±0.3 SE) of ammonia oxidized by *N. viennensis* grown on 2 mM NH_4_^+^. Although dissolved inorganic N (DIN) concentrations (sum of [NH_4_^+^] and [NO_2_^–^]) were relatively constant over the course of ammonia oxidation, we recovered only 81.9% (±1.5 SE) of the initial DIN by the end of incubation of *N. adriaticus*, and 94.7% (±3.4 SE) and 90.7% (±1.1 SE) of *N. viennensis* grown on 1 mM NH_4_^+^ or 2 mM NH_4_^+^, respectively. In *N. adriaticus* cultures, assimilation of N by contaminant bacteria likely did not contribute substantially to the lower ε_*RS*_ relative to ε_*CP*_, due to the high enrichment level of the culture (95%) at the time of the experiment, and the fact that ε_*RS*_ of *N. adriaticus* was similar to that of *N. viennensis* in pure culture. In addition, the ^15^N-enrichment of *N. viennensis*’ biomass shows that AMO preferentially, and primarily, reacts on pseudo-periplasmatic NH_3_, causing ^15^N-enrichment of the residual ammonia, which is subsequently assimilated into biomass. We thus propose that under substrate replete conditions, the observed isotope effects of ε_*RS*_ of 31.9–33.1‰ primarily reflect the kinetic isotope effect of the AMO-catalyzed reaction, modified by the NH_4_^+^/NH_3_ equilibrium isotope effect (19.2‰; Hermes et al., 1985) and decreased by the contribution of the lower kinetic isotope effect of NH_4_^+^ assimilation for anabolic purposes (4–27‰; Hoch et al., 1992). Moreover, it should be noted that some ammonia oxidizers use distinct pathways of NH_4_^+^ assimilation, even among just AOA, which may contribute to different kinetic isotope effects. For instance, some members of the AOA genus *Candidatus* Nitrosocosmicus appear to assimilate NH_4_^+^ via glutamate synthase (GOGAT), whereas all other known AOA use the glutamate dehydrogenase (GDH) pathway (Alves et al., 2019).

Despite these potential isotope fractionation effects on the RS level, a higher ε_*CP*_ relative to ε_*RS*_ may also result from accumulation of metabolic intermediates, allowing for at least a second ^15^N isotope fractionation step to be observed. First, accumulation of NH_2_OH, or any other intermediate, has not been observed in AOA cells, and the coupled activities of AMO and hydroxylamine oxidoreductase (yet unknown in AOA) are assumed to maintain NH_2_OH at low steady-state concentrations. Therefore, isotope effects associated with NH_2_OH oxidation should have a limited impact on δ^15^NO_2_^–^. Second, any process that adds an additional isotope fractionation step, either prior, or subsequent, to NO_2_^–^ formation, such as the production of the gases NO and N_2_O, may result in an under- or overestimation of the kinetic isotope fractionation factor. The effect of N_2_O and NO production on δ^15^NO_2_^–^ will depend on their formation pathways, and respective isotope effects and by-product yields. In the *N. viennensis* culture grown on 2 mM NH_4_^+^, we measured cumulative N_2_O at the end of the incubation, which represented 0.5% (±0.01 SE) of the NH_4_^+^ oxidized, or 3.5% (±0.4 SE) of the “missing” N pool. N_2_O yields of AOA are generally low. For example, *N. viennensis* has been shown to produce N_2_O at rates of about 0.1% of those of ammonia oxidation when grown on 1 mM NH_4_^+^/NH_3_ (Stieglmeier et al., 2014b), whereas the marine AOA *N. maritimus* SCM1 produces even less (0.002–0.03%; Löscher et al., 2012). Here, the cumulative N_2_O of the *N. viennensis* culture had a δ^15^N of −38.1‰ (±0.3 SE) (Figures 1F, 3), which was more ^15^N-depleted than previously observed for AOA enrichment cultures. AOA have been shown to produce N_2_O with δ^15^N signatures ranging between −35 and −13‰ in soil enrichment cultures and −9‰ in marine enrichment cultures (Santoro et al., 2011; Jung et al., 2014), while N_2_O produced by AOB tends to have lower δ^15^N, ranging between −66‰ (*Nitrosomonas europaea*; Yoshida, 1988) and −10‰ (*Nitrosomonas marina*; Frame and Casciotti, 2010). Furthermore, by-products that are more ^15^N-depleted than the main product of ammonia oxidation (i.e., NO_2_^–^) would decrease the apparent kinetic isotope effect of AMO (ε_*CP*_), instead of increasing it. Using an isotopic mass balance approach, we calculated that the missing N pool (i.e., the NH_4_^+^ taken up which was not oxidized to NO_2_^–^), would need to have a δ^15^N of −18.5‰ (±1.7 SE) in order to account for the difference in isotope fractionation between ε_*RS*_ and ε_*CP*_ (Table 2). Therefore, the δ^15^N_2_O signature of −38.1‰ cannot explain the observed large isotope fractionation based on δ^15^NO_2_^–^, since the N_2_O produced would need to be a larger contributor to the “missing” N pool, as well as to be ^15^N-enriched relative to NO_2_^–^.

**TABLE 2:** Nitrogen pools for *N. viennensis* culture grown on 2 mM NH_4_^+^.

	**N pool (μM)**	**δ^15^N of N pool (‰)**	**Percent of missing N pool**	**Percent of ammonia oxidized**
Missing	204.4 (±25.2)	−7.6 (±5.2)		
Biomass	38.8 (±3.4)	10.1 (±0.1)	22.8 (±4.1)	3.1 (±0.3)
N_2_O-N	6.5 (±0.2)	−38.1 (±0.3)	3.5 (±0.4)	0.5 (±0.1)
Unaccounted	139.2 (±27.1)	−18.5 (±1.7)	73.7 (±4.5)	

*The missing N pool is N that was not present at NH_4_^+^ and NO_2_^–^ at the last sampling time point when biomass and N_2_O was collected for isotope analysis. We used a mass balance approach to calculate δ^15^N of the missing and unaccounted N pool. Standard errors are given in parentheses.*

Nitric oxide is an important intermediate in the ammonia oxidation pathway, particularly in that of AOA. Unlike in AOB, NO is a necessary co-reactant for the oxidation of NH_2_OH to NO_2_^–^ in AOA, despite being produced in relatively small amounts (Kozlowski et al., 2016). Although the δ^15^N signature of NO produced by AOA has not yet been determined, Yoshida (1988) found that NO produced during nitrification by *N. europaea* had a δ^15^N between 0 and +20‰. The production of such ^15^N-enriched NO could significantly contribute to the observed overestimation of ε_*CP*_ in AOA.

## Conclusion

In conclusion, our results show that, under non-limiting substrate conditions, the ε_*AMO*_ of two phylogenetically and ecologically distinct AOA strains was 31.9–33.1‰ based on δ^15^NH_4_^+^, whereas the more commonly estimated ε_*AMO*_ based on δ^15^NO_2_^–^ was higher (37.7–40.1‰). Thus, NH_4_^+^ assimilation, but not N_2_O production, significantly affected the isotope fractionation factor of AMO estimated for *N. viennensis* (Figure 3). Although the potential role of NO in this context remains to be tested, isotopic analysis of this molecule is difficult and therefore future measurements of ε_*AMO*_ may rely on coupled estimates from δ^15^NH_4_^+^ and δ^15^NO_2_^–^.

## Data Availability Statement

The raw data supporting the conclusion of this article have been deposited at DRYAD (doi: 10.5061/dryad.0gb5mkkz1).

## Author Contributions

WW designed the study. MMo, RA, BB, MMe, MS, LJ, SR, and MW performed the experiments. MMo, RA, BB, MS, and LJ analyzed the data. GH and CS provided the resources and strains. MMo, RA, and WW wrote the manuscript with contributions from all co-authors.

## Conflict of Interest

The authors declare that the research was conducted in the absence of any commercial or financial relationships that could be construed as a potential conflict of interest.
